# Virologic Failure, Clinical Characteristics, and Common Viral Mutations in HIV Patients From Southwestern Colombia: A Nested Case-Control Study

**DOI:** 10.7759/cureus.68530

**Published:** 2024-09-03

**Authors:** María E Tello-Cajiao, Leonardo Montero, Reynaldo Carvajal Ortiz

**Affiliations:** 1 Internal Medicine, Grupo Interinstitucional de Medicina Interna (GIMI1) Universidad Libre, Cali, COL; 2 Infectious Diseases, Clinica TodoMed, Cali, COL; 3 Epidemiology and Biostatistics, Grupo Interinstitucional de Medicina Interna (GIMI1) Universidad Libre, Cali, COL

**Keywords:** hiv, sustained viral suppression, public health, opportunistic infections, antiviral drug resistance, cd4-positive t-lymphocytes, epidemiology

## Abstract

Introduction

Virologic failure due to antiretroviral drug resistance is a threat to efforts to control the human immunodeficiency virus (HIV) epidemic. Understanding the factors that influence the genetic and clinical expression of drug resistance is fundamental for infection control.

Methods

A nested case-control study was conducted on a cohort of adult HIV patients between 2016 and 2022. The cases were defined as patients with a confirmed diagnosis of virologic failure due to drug resistance, as indicated by a viral genotype result. The control group consisted of patients who had not experienced virologic failure or undergone any documented changes to their antiretroviral treatment. The incidence of virologic failure over a defined period was calculated. The characteristics of each group were documented in frequency tables and measures of central tendency. To identify risk factors, multiple logistic regression models were employed, and post hoc tests were conducted. All calculations were performed with 95% confidence intervals, and p-values less than 0.05 were considered significant.

Results

The incidence of virologic failure over the seven-year study period was 9.2% (95% CI: 7.5-11.2%). Low CD4 T-lymphocyte count (≤200 cells/mm³) at diagnosis (adjOR 14.2, 95% CI: 3.1-64.5), history of opportunistic infections (adjOR 3.5, 95% CI: 1.9-6.4), and late enrollment into an HIV program after diagnosis (>1 year) (adjOR 9.2, 95% CI: 3.8-22.2) were identified as independent predictors of virologic failure. The drugs with the highest rates of resistance were nevirapine (84.6%), efavirenz (82.4%), emtricitabine (81.3%), lamivudine (81.3%), and atazanavir (6.6%). The most prevalent major mutations identified were K103N, M184V, and M46I/M. Approximately 50% of the secondary mutations were identified in protease regions.

Conclusions

The incidence of virologic failure was low in the study population. The identified risk characteristics allow for the prediction of the profile of patients susceptible to failure and for the early optimization of treatment regimens.

## Introduction

Human immunodeficiency virus (HIV) remains a major public health problem. Since the onset of the epidemic, it is estimated that 85.6 million individuals have been infected, with at least 40 million deaths attributed to acquired immunodeficiency syndrome (AIDS)-related illnesses [1,2]. In this context, virologic failure due to acquired resistance to antiretroviral drugs represents a significant challenge to efforts to control and eradicate the infection. Consequently, research in this area is of critical importance. The World Health Organization (WHO) defines virologic failure as a persistently detectable viral load greater than 1,000 copies/mL in two consecutive measurements within three months after at least 24 weeks of antiretroviral therapy (ART) [3]. Nevertheless, with enhancements in laboratory test efficacy, the cut-off points for the definition have been diminished [4].

The occurrence of virological failure can be attributed to a number of factors. From a virological perspective, wild-type HIV strains exhibit superior "viral fitness" and tend to predominate over resistant variants [5]. Therefore, the attainment of viral suppression objectives is anticipated when therapeutic drug concentrations are sustained. However, intermittent exposure to treatment creates selective pressure for the emergence of resistant variants, which eventually become the majority of the viral population (acquired resistance) [6]. Moreover, the high error rate of the enzyme reverse transcriptase (RT) enhances the probability of resistance mutations accumulating. During the process of viral replication, the enzyme RT is unable to effectively correct errors in the transcription of proviral DNA, which can contribute to the emergence of resistant variants. These situations have several implications for antiretroviral drug resistance in clinical practice. Primary mutations increase drug resistance at the expense of replication ability, while secondary mutations affect resistance to a lesser extent but improve viral replication [7]. It is therefore crucial to perform genotyping tests and identify the risk factors present in the patient in order to define the optimal therapeutic strategy. This study examined the occurrence and contributing factors associated with virological failure, as well as the most prevalent resistance mutations in HIV-infected adult patients treated within an HIV program located in southwestern Colombia.

## Materials and methods

Study design and patient selection

A nested case-control study was conducted in a cohort of HIV-diagnosed adult patients attending a health facility in Cali, Colombia, between 2016 and 2022. A case was defined as any patient on ART with a sustained viral load greater than 50 copies/mL and an available viral genotype result. Controls were defined as all patients on ART with no history of previous virologic failure or documented changes in their therapy since the diagnosis of infection. Patients with no treatment, an unknown ART start date, treatment changes due to poor adherence, intolerance, adverse effects, drug administration problems, or less than 180 days of antiretroviral exposure were excluded.

Variables of interest and data collection

Sociodemographic and epidemiologic characteristics, such as age, sex, ethnicity, health insurance, membership in key populations (e.g., LGBTQIA+), and likely mode of transmission, were included. Clinical characteristics included history of comorbid and opportunistic infections, clinical stage at diagnosis, days from diagnosis to HIV program entry and ART initiation, CD4 T-lymphocyte count, viral load, and vital status at last follow-up. Data were collected from the annual HIV cohort registry reported to the Ministry of Health. This study was approved by the Ethics and Research Committee of TodoMed and the Universidad Libre (Act No. 1 CI-02-21-08; February 21, 2024).

Data analysis

The cumulative incidence of the diagnosis of virologic failure in each study year and time cluster was calculated. For categorical variables, absolute and relative frequency tables were constructed and compared using the Chi-square test or Fisher's test (when appropriate). Quantitative variables were described with the median (Me) and interquartile range (IQR), as they all had a skewed distribution, and were compared using the Mann-Whitney U test. Variables with a p-value ≤0.10 in the bivariate analysis were included in the modeling. To reduce the possibility of overfitting in the final model, collinearity between the selected variables was analyzed using Spearman's test.

Multivariate logistic regression models were carried out using the backward system. Adjusted odds ratios (ORs) and Wald tests were estimated. Due to differences in degrees of freedom and the number of observations for some variables, the Akaike information criterion was used to select the most parsimonious model. Goodness-of-fit tests were performed on the final model (Hosmer-Lemeshow test and receiver operating characteristic curve (ROC)). p-values <0.05 were considered significant. All calculations were performed with 95% confidence intervals (95% CI). Stata software, version 15 (StataCorp LLC, College Station, TX, USA), was used for analysis.

## Results

**Figure 1:**
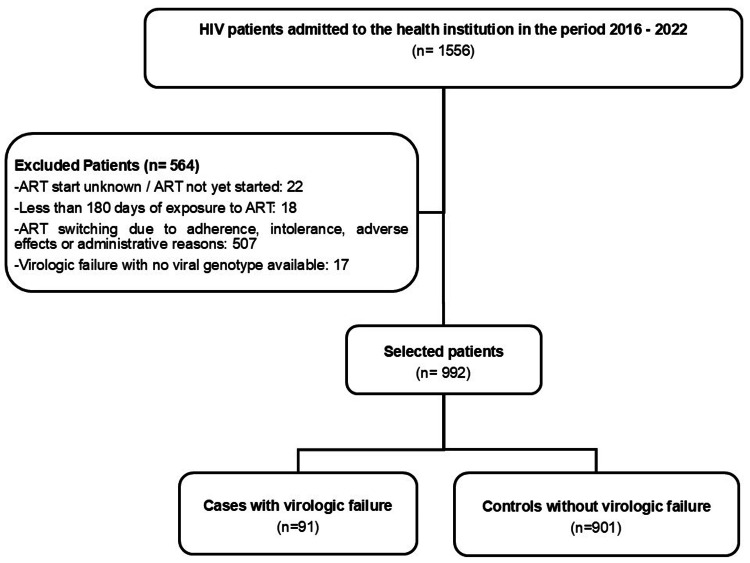
Diagram of the selection of the study population HIV: Human immunodeficiency virus; ART: Antiretroviral therapy

During the period from 2016 to 2022, we identified 1,556 adult patients admitted to the health center for HIV care. According to the selection criteria, 91 cases of virologic failure and 901 controls, with no history of virologic failure and no known changes in their ART regimen, were enrolled (Figure 1).

Incidence of virologic failure

In each year of follow-up, the median number of HIV patients admitted to the health center was 135 (IQR: 113-174 patients), with a median of 13 cases of virologic failure diagnosed per year. The estimated cumulative incidence of virologic failure over the seven-year period was 9.2% (95% CI: 7.5-11.2%). In the group of patients with virologic failure, 20.8% were diagnosed in the first 12 months of ART exposure, with a median time of 33 months (IQR: 16-68 months).

Characteristics and clinical differences between groups

Most subjects were male (65.5%, n = 650), with a median age of 35 years (IQR: 29-46 years). Mixed ethnicity and subsidized health insurance were predominant in the population (98.9% and 97.3%, respectively). Of the patients, 31.5% belonged to the LGBTQIA+ community. At least 10% of subjects used a psychoactive substance. There were seven sex workers and two homeless people in the control group. The most frequent probable mechanism of transmission was sexual (97.5%, n = 968). Nearly half of the patients had a history of comorbid or opportunistic infections (49.3%, n = 489). The proportion of opportunistic infections was significantly higher in the cases (51.6%, n = 47, p-value = 0.000). These included tuberculosis, cerebral toxoplasmosis, *Pneumocystis jirovecii*, cytomegalovirus, herpesvirus, and candidiasis. The most common comorbid infection in both groups was syphilis (33.06%, n = 328).

At the time of diagnosis, 37.1% (n = 368) of patients were in clinical stage 2 according to the CDC (Centers for Disease Control and Prevention, 2014) classification. However, most cases were diagnosed at clinical stage 3 (41.7%, n = 38). CD4 T-lymphocyte count was significantly lower in patients with virologic failure both at diagnosis (Me: 114 cells/mm³, p-value = 0.000) and at ART initiation (Me: 112 cells/mm³, p-value = 0.000). Median viral load was not statistically different between groups. After HIV diagnosis was confirmed, cases required more days to enroll in an HIV program and initiate ART than controls (Me: 111 cells/mm³ vs. 65 days). The documented mortality in the program was 1.31%, with no differences between groups. Approximately 10% of patients were lost to follow-up, with no significant differences between the groups (Table 1).

**Table 1:** Characteristics and clinical differences between selected cases and controls (n = 992) Me: Median; IQR: Interquartile range; CDC: Centers for Disease Control and Prevention; ART: Antiretroviral therapy

Characteristics	Total (n = 992)	Controls without virologic failure (n = 901)	Cases with virologic failure (n = 91)	p-value
Age in years, Me (IQR)	35 (29-46)	35 (28-46)	37 (41-48)	0.064
Sex male, n (%)	650 (65.52)	597 (66.26)	53 (58.24)	0.125
Subsidized health insurance, n (%)	965 (97.28)	874 (97.00)	91 (100)	0.165
Ethnicity, n (%)				
Mestizo	981 (98.89)	890 (98.78)	91 (100)	0.612
Indigenous and Afro	11 (1.11)	11 (1.22)	-	
Key populations, n (%)				
LGBTQIA+ community	312 (31.45)	293 (32.52)	19 (20.88)	0.159
Psychoactive drug users	108 (10.89)	97 (10.77)	11 (12.09)	
Sex workers and homeless people	9 (0.90)	9 (0.99)	-	
Likely mechanism of transmission, n (%)				
Sexual	968 (97.58)	879 (97.56)	89 (97.80)	1
Other	24 (2.42)	22 (2.44)	2 (2.20)	
Comorbid and opportunistic infections, n (%)				
Syphilis	328 (33.06)	291 (32.30)	37 (40.66)	0.106
Tuberculosis	100 (10.08)	76 (8.44)	24 (26.37)	0
Cerebral toxoplasmosis	45 (4.54)	31 (3.44)	14 (15.38)	0
Pneumocystis jirovecii	41 (4.13)	26 (2.89)	15 (16.48)	0
Cytomegalovirus	41 (4.13)	31 (3.44)	10 (10.99)	0.003
Herpes virus	39 (3.93)	31 (3.44)	8 (8.79)	0.021
Hepatitis B	38 (3.83)	32 (3.55)	6 (6.59)	0.151
Candidiasis	34 (3.43)	25 (2.77)	9 (9.89)	0.002
Cryptococcosis	17 (1.71)	13 (1.44)	4 (4.40)	0.062
Histoplasmosis	8 (0.81)	7 (0.78)	1 (1.10)	0.538
Hepatitis C	5 (0.50)	5 (0.55)	-	1
Clinical stage CDC at diagnosis, n (%)				
Stage 1	206 (20.77)	204 (22.64)	2 (2.20)	0
Stage 2	368 (37.10)	347 (38.43)	21 (23.08)	
Stage 3	264 (26.61)	226 (25.03)	38 (41.76)	
Stage unknown	154 (15.52)	124 (13.76)	30 (32.97)	
CD4 T-lymphocyte count (cells/mm^3^) and viral load (copies/mL), Me (IQR)				
CD4 T-lymphocytes at diagnosis	327 (159-496)	340 (176-513)	114 (56-321)	0
Viral load at diagnosis	21411 (2834-110645)	20878 (2834-107364)	26865 (1661-120515)	0.847
CD4 T-lymphocytes at ART initiation	323 (154-490)	337.5 (171-504)	112 (56-296)	0
Viral load at ART initiation	19340 (2372.5-100196)	18651.5 (2490-98992)	24748 (1661-120515)	0.657
Days from diagnosis to HIV program enrolment and ART initiation, Me (IQR)				
At HIV program enrollment	42 (22.5-93.5)	40 (22-83)	111 (44-1168)	0
At ART initiation	46 (22-89.5)	44 (22-83)	65 (24-163)	0.02
Deceased, n (%)	13 (1.31)	10 (1.11)	3 (3.30)	0.185

Factors associated with virologic failure in the population

**Figure 2:**
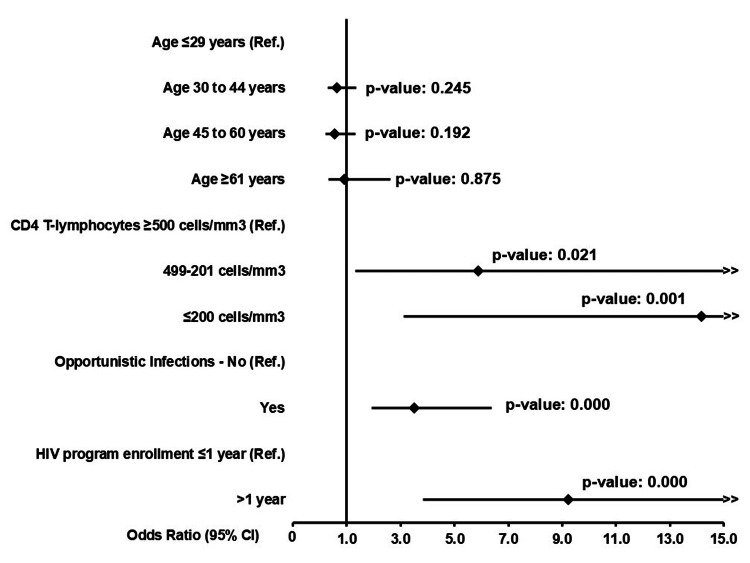
Factors associated with the diagnosis of virologic failure in the study population (n = 992)

Clinical stage at diagnosis, CD4 T-lymphocyte count at ART initiation, and days from diagnosis to ART initiation had high Spearman coefficients and were not included in the final model. Multiple logistic regression showed that a low CD4 T-lymphocyte count (≤200 cells/mm³) at diagnosis (adjOR 14.2, 95% CI: 3.1-64.5), a history of opportunistic infections (adjOR 3.5, 95% CI: 1.9-6.4), and late entry into an HIV program after diagnosis (>1 year) (adjOR 9.2, 95% CI: 3.8-22.2) significantly increased the odds of virologic failure in the study population. There was even an increase in the odds of virologic failure as the CD4 T-lymphocyte count decreased. Age did not show an association with virologic failure in the model (Figure 2). The pseudo-R² of the regression was low (0.1822), but the final model selected had a good fit (ROC: 0.8216, Hosmer-Lemeshow test p-value = 0.295). The post-hoc power to estimate the incidence of virologic failure was 100%, using as a frame of reference a known rate of 12.4% annual virologic failure according to the latest available data from the Colombian Ministry of Health [8].

Antiretroviral drug resistance and common mutations

The non-nucleoside reverse transcriptase inhibitors (NNRTIs) with the highest number of resistance reports were nevirapine (NEV) (84.6%, n = 77) and efavirenz (EFV) (82.4%, n = 75). The major mutation in this family was K103N, which appeared in 43% of the genotypes. Among the nucleoside reverse transcriptase inhibitors (NRTIs), emtricitabine (FTC) and lamivudine (3TC) were the antiretrovirals with the highest rate of resistance (81.3% for both). The most common primary mutation was M184V, with a frequency of 40% in this group. Protease inhibitors (PIs) had the lowest reported frequency of resistance. Atazanavir (ATV) had the highest frequency of resistance in the group (6.6%, n = 6), and the main primary mutation in this family was M46I/M (11%). A total of 135 secondary viral mutations were found: 52.6% in protease (PR) regions (n = 71) and 47.4% in RT regions (n = 64). The most frequent PR mutations were L63P (n = 41), E35D (n = 29), V77I (n = 26), I93L (n = 24), and I62V (n = 22). The most common secondary mutations in RT were 211K (n = 54), F214L (n = 14), V118I (n = 12), S68G (n = 9), and A98S (n = 8).

## Discussion

The variations in the epidemiological profile of virologic failure can be attributed to changes in its operational definition, which have resulted from improvements in diagnostic test performance. The data indicate that the cumulative incidence of virologic failure in this population was 9.2% (95% CI: 7.5-11.2%). This frequency appears to be relatively low compared to that observed in other studies, which, using the WHO definition, have estimated virologic failure rates as high as 20%. This is particularly the case in the context of subtype 1 infection. It should be noted, however, that these estimates vary by geographic region [5,9-11]. It is possible that the data have been underestimated, given that only patients with available viral genotype results were included. Nevertheless, data from the Colombian Ministry of Health, which employs a viral load cut-off point as rigorous as that utilized in this study, indicate a viral suppression failure rate of just 12.4% [8]. Other studies conducted with the Colombian population reveal that the incidence of virological failure ranges between 7.8% and 20%, with treatment failure rates reaching up to 30% [12,13]. It is of great importance to consider the epidemiology of virologic failure, as the probability of selecting mutations that drive acquired drug resistance is significantly reduced when viral RNA levels are persistently suppressed, ideally below the lower limit of detection of current assays [14-16].

There are numerous potential causes for virologic failure, but low adherence to the prescribed therapy is arguably the most significant risk factor. A lack of adherence has been demonstrated to increase the odds of virologic failure by a factor of 6-12 [13]. Drug intolerance, side effects, and toxicity are critical to the decision to discontinue treatment, and it is therefore imperative to identify these factors in HIV programs [17,18]. Additional factors associated with virologic failure include male sex, low hemoglobin at the initiation of therapy (<12 mg/dL), advanced clinical stage, history of psychoactive substance use, and others [12,19,20]. The results of this study indicate that a low CD4+ T cell count at initial diagnosis (particularly below 200 cells/mL), a history of opportunistic infections, and late enrollment in an HIV program (>1 year) were identified as independent risk factors for virologic failure in the study population, in line with the findings of other researchers in the field [6,19].

Opportunistic infections represent one of the most consistent elements in the association with virologic failure. Their presence imposes an additional workload on the immune system, contributing to the depletion of the CD4 cell population and creating an environment conducive to viral replication [20]. Likewise, low lymphocyte levels increase the likelihood of developing opportunistic infections, particularly those with a predominantly cell-mediated immune response, such as tuberculosis. Late enrollment in an HIV program leads to serious consequences. From a clinical perspective, delayed entry into a program allows the disease to progress to more advanced stages. As evidenced by the data, up to 42% of cases were classified as stage 3 at the time of diagnosis. In this regard, research has identified two key reasons for delaying contact with the HIV program: self-perception of feeling healthy and fear of discrimination [21]. Additionally, this situation represents a significant challenge for public health, given that early diagnosis and access to health care are essential for infection control [22].

In the analysis of drug resistance, the highest proportion was recorded with regimens including FTC, 3TC, NVP, and EFV. These drugs have been shown to have a low barrier to resistance, and for some of them, a single mutation is sufficient to induce resistance [23]. In contrast, PIs exhibited the lowest incidence of resistance in the study population. These agents have the advantage of a high barrier to resistance and are often utilized in patients who have not responded to an initial regimen. Notwithstanding their strengths, it has been observed that in individuals with CD4 T cell counts below 350 cells/mL or with AIDS-defining events at diagnosis, mutations that result in the failure of this drug class may accumulate, both in treatment-experienced and treatment-naïve patients. This suggests the possible irreversible fixation of resistance mutations [23]. In this context, it is of paramount importance to closely monitor the emergence of secondary mutations, as they frequently occur in protease regions. The accumulation of these mutations could potentially reduce the genetic barrier to drugs targeting this enzyme [24]. To illustrate, the L63P protease mutation, which was the most prevalent in this study, does not exert an effect on any PI individually. However, it is identified in viruses lacking prior exposure to these drugs and may be more prevalent in patients who have experienced failure with PI-containing regimens [25].

The review of virological failure due to acquired drug resistance and the most frequent resistance mutations over the seven years included in this study may reflect more traditional antiretroviral regimens. This has significant clinical and public health implications, as data from the Ministry of Health indicate that the most commonly used antiretroviral drugs in Colombia are TDF, 3TC, and FTC [8], which presented the highest frequencies of resistance in the present study. The current first-line treatment recommendations include integrase inhibitors in combination with NRTIs, due to their efficacy, safety, tolerability, and high resistance barrier [4]. It would be prudent for HIV programs in the country to evaluate the benefits of adhering to current recommendations and discuss these with their patients.

A potential limitation of the study was its retrospective design, which could introduce information bias. However, the data typists have extensive experience in the management of HIV patients, and data were rechecked when necessary. Another limitation was the low pseudo-R² of the regression, indicating the exclusion of variables that could more effectively explain the observed data variability. This was anticipated, given that low adherence represents the primary risk factor for virological failure and was not included in the analysis for this cohort of patients. Nevertheless, the study offers a valuable contribution to the local knowledge base on other clinically relevant variables in the context of virological failure. The primary strength of the study was the achievement of a robust estimate of the incidence of virological failure in the population, with a post hoc power of 100%. However, we advise caution when interpreting this estimate, as there is a possibility of an underlying underestimation of cases, given that only patients with available genotype results were included in the analysis.

## Conclusions

The occurrence of virological failure due to acquired resistance to antiretroviral drugs appears to be a relatively low-frequency phenomenon within the studied population. By identifying the factors associated with its occurrence, such as a low CD4 count, opportunistic infections, and delayed entry into the HIV program, healthcare professionals can anticipate the risk to patients and implement early action plans. In light of the above, surveillance of drug resistance and its associated mutations is critical to optimize treatment regimens for high-risk populations. Further research is required in this field to evaluate the impact of current integrase inhibitor-based regimens on the epidemiology of virological failure and its consequences.
